# The association of factor VIII activity levels with bleeding and quality of life in haemophilia a: findings from the European CHESS II study

**DOI:** 10.1186/s13023-025-03699-z

**Published:** 2025-06-03

**Authors:** Cheryl Jones, Yunchou Wu, Nana Kragh, Linda Bystrická, Amanda Wilson, Tom Burke

**Affiliations:** 1 HCD Economics, Brook Street, Cheshire, Knutsford, WA16 8GP UK; 2 Sobi, Stockholm, Sweden; 3 Sanofi, Cambridge, MA 02141 USA

**Keywords:** Annual bleeding rate, Factor VIII level, Hemophilia A, Health-related quality of life, Quality of life, Haemophilia

## Abstract

**Background:**

Haemophilia A is a rare disorder leading to excessive bleeding, resulting from a deficiency in circulating clotting factor VIII. While we know that raising factor VIII levels for extended periods of time would reduce the frequency of injections required and provide more consistent bleed protection, the relationship between factor activity levels (FALs), clinical outcomes, and health-related quality of life (HRQoL) for people with haemophilia A (PwHA) has not been well characterised.

**Objectives:**

This study explored the relationship of FALs with annual bleeding rate (ABR) and HRQoL in PwHA.

**Methods:**

This retrospective, cross-sectional study used data from the CHESS II study (2018–2020) including men aged ≥ 18 years with haemophilia A in Europe. Physicians provided patient characteristics and clinical outcomes from the medical records. Patients completed a questionnaire with HRQoL (EQ-5D) and socio-economic information. PwHA receiving on-demand treatment who had baseline factor VIII levels < 40 IU/dL and no inhibitors were included. Regression models explored FALs with ABR and HRQoL controlling for age, BMI, and presence of blood-borne viruses.

**Results:**

A total of 403 PwHA were included (167 provided HRQoL responses). Mean age was 37 years. Mean baseline FAL was 7.0 IU/dL and mean ABR was 2.3 (SD, 2.64). A negative binomial model showed for every 1% increase in FVIII levels, ABR decreased by 3.9% (0.09 events; *P* < 0.001). A tobit model showed that every 1% increase in FVIII levels was associated with an increase of 0.0054 points in mean EQ-5D index score (*P* < 0.001).

**Conclusions:**

This study offers tangible estimates of how higher FALs may relate to lower ABR and elevated HRQoL for PwHA.

**Supplementary Information:**

The online version contains supplementary material available at 10.1186/s13023-025-03699-z.

## Background

Haemophilia A (HA) is a rare genetic disorder characterised by excessive bleeding caused by insufficient or absent clotting factor VIII. People with HA (PwHA) can be classified as having mild (> 5–40% of normal factor VIII levels), moderate (1–5%), or severe (< 1%) disease [1]. A bleeding prevention strategy comprised of routine prophylactic administration of factor VIII or non-factor therapy is the standard of care for PwHA, particularly those with moderate or severe HA, though on-demand treatment of bleeding events is used when necessary or preferred by the patient [2]. Repeated bleeding events are associated with chronic joint disease and pain, and the related morbidity worsens with severity as bleeding events are more common with severe HA than with mild HA [3, 4].

The lifelong nature of a HA management regimen imposes a notable burden on PwHA and their families, and spontaneous bleeding events can still occur despite continuous treatment [5–7]. These breakthrough bleeding events ultimately cause progressive joint damage that has substantial effects on health-related quality of life (HRQoL), work productivity, and costs to patients, their families, and society [8–10].

Despite the advances proffered by modern prophylactic treatment regimens to reduce morbidity and mortality, the pursuit of a functional cure for HA persists in clinical research [11]. Recently developed extended half-life factor products have shown the capability to raise factor levels to within normal ranges for the majority of a week with once weekly dosing. This can help improve clinical outcomes as well as HRQoL by reducing the occurrence of bleeding events and the number of factor injections required [12, 13]. The relationship between factor activity levels (FALs) and clinical and HRQoL outcomes, however, has not been well characterized in the literature, particularly among people with mild or moderate HA.

A previous study investigated the relationship between annual bleeding rate (ABR) and FALs in males with haemophilia B in an analysis of the Cost of Haemophilia in Europe: a Socioeconomic Survey (CHESS) study [14]. Their model reported that every 1% increase in FALs was associated with a decrease in the average ABR by 0.08 events, providing a tangible measurement of the rate at which elevated FAL reduced bleeding events in people with haemophilia B. A follow-up study by the same group also reported statistically significant, non-linear relationships between FALs and HRQoL, where increases in FALs were associated with increases in HRQoL [15]. In the absence of such investigations for PwHA, this study sought to build on previous work to explore the relationship of FALs with ABR and HRQoL in PwHA participating in the CHESS study. This information is required to firstly validate and compare to pre-existing findings, which were the first of their kind, and secondly to expand the research into a new population to underpin the economic evaluation of novel therapeutic options specific to that patient population, as required by health technology assessment bodies.

## Methods

### The CHESS study design, and data overview

This retrospective, cross-sectional study used data from the CHESS II study, conducted in 2018–2020, including European PwH (≥ 18 years) with haemophilia A or B in France, Germany, Spain, Italy, the United Kingdom, Denmark, The Netherlands, and Romania. Design and methods of the CHESS study have been reported previously [16, 17].

CHESS study data were collected via 2 questionnaires designed specifically for haematology specialists and people with haemophilia. Physicians completed web-based case record forms (CRFs) with patient demographic and clinical characteristics as well as clinical outcomes from the medical records. Patients completed a Patient Public Involvement Engagement (PPIE) questionnaire with additional socio-economic information [16]. Eligible PwHA who chose to participate provided informed consent. The study protocol was approved by the Research Ethics Sub Committee of the Faculty of Health and Social Care at the University of Chester and the study was carried out according to the corresponding ethical guidelines [16].

### Participants and eligibility criteria

Physician participants had to be actively practicing haematology specialists in the countries of interest (with the exception of France, where Haemophilia Care Providers could also participate) for ≥ 3 years with a minimum caseload of ≥ 4 males with haemophilia per month. Eligible physicians were invited to retrospectively enrol a target of 4 of their patients with haemophilia and to retrospectively complete the CRFs with information extracted from the medical records over the previous 12 months. Physicians were recruited via a fieldwork agency using a convenience sampling method [16]. In order to minimize the risk of selection bias, physicians were encouraged to recruit the next eligible patients with scheduled consultations regardless of the reason for their consultation [16].

Additional patient eligibility criteria were applied for this study’s specific objectives. Only persons who had baseline factor VIII levels < 40 IU/dL, a record of baseline Factor VIII (FVIII) and without a current inhibitor were included. PwHA receiving prophylactic treatment regimens were excluded. People with a particularly high ABR (≥ 100 bleeds/year) and/or high consumption of factor replacement therapy (> 60,000 IU/year) were also excluded.

### Variables and outcomes

FALs and ABR data were collected by the physicians along with patients’ demographic information in the CRFs. HRQoL data were collected from PwHA in the PPIE. All data in this study were collected during 2018–2020 and anonymized to ensure protection of personal information.

Demographic and clinical characteristics included age, ethnicity, height, weight, body mass index (BMI), smoking status, disease severity, number of target joints [1] and number of problem joints [18]. Clinical variables included the assay used to measure FVIII level, comorbidities (i.e., blood-borne viruses), consultation history (frequency of contact and visits with the haematologist and other specialists), hospital admissions, factor replacement therapy, age at initiation of factor replacement therapy, and concomitant medications. In addition to HRQoL questionnaire responses, PwHA reported their employment status, highest level of education, household income, HA severity (mild, moderate or severe), frequency of bleeding events, pain, number of injections, and compliance/adherence to HA treatment.

Baseline FALs were taken from the medical record for each patient (extracted by the physician). ABR was reported by the physician as the number of bleeding events in the 12 months prior to enrolment in the study. Bleeding events included the number, timing and circumstances of the event, and were stratified by severity (major, minor bleed), location (i.e., joint), and type (spontaneous, trauma-related).

Patient-reported HRQoL included self-reported health state using the 5-level EQ-5D (EQ-5D-5 L; euroqol.org). The EQ-5D-5 L has 5 domains (mobility, self-care, performance of usual activities, pain/discomfort and anxiety/depression) evaluated according to 5 levels of severity (“no problems,” “slight problems,” “moderate problems,” “severe problems,” or “extreme problems”). Health state index utility scores across all domains ranged from zero to 1, where zero indicated a health status rating equivalent to “death” and 1 indicated “perfect health.” Scores less than zero (“worse than dead”) are possible in the EQ-5D. The domains of the measure are linked to a value set in order to derive a single summary index value of health status. The value set provides weights for each health state based on the preferences of the general reference population of a particular country or region. EQ-5D-5 L value sets are available for several countries; this study used the United Kingdom 5 L value set derived from mapping (crosswalk) the Eq. 5D-3 L value set to the Eq. 5D-5 L value set [19]. The mapped Eq. 5D-5 L UK value set was applied to all countries for analysis to enable comparison.

### Statistical analysis

Demographic and clinical characteristics were summarized using descriptive statistics and measures of central tendency. Scatterplots and Spearman’s rank correlation analyses were used to assess the relationships between baseline FALs and ABR, and FALs and HRQoL. Spearman’s test describes the strength and significance of the relationship between pairs from ‘very weak’ (0-0.2), to ‘very strong’ (0.8-1.0). Regression models used FALs as the independent variable and ABR and HRQoL as the dependent variables, controlling for known risk factors of age, BMI, and presence of blood-borne viruses (human immunodeficiency virus [HIV], hepatitis B virus [HBV], and hepatitis C virus [HCV]) [20]. Covariate selection was also aligned with previous studies modelling the relationship between FAL and ABR [15, 21].

The model with the best goodness-of-fit was selected using Akaike Information Criterion (AIC), where the lowest value represents the best fit. For the models assessing the relationship between FALs and ABR, it was noted that model bleed (count) data can pose difficulties due to a large proportion of individuals experiencing zero bleeds and a minority experiencing a high number of bleeds, resulting in a highly right skewed distribution. Therefore, generalized Poisson regressions (Poisson, negative binomial, zero-inflated Poisson, zero-inflated negative binomial) and generalized linear models with log-link were used to explore the association between changes in FAL and ABR, adjusting for age, BMI, HIV, HBV, and HCV. For the models assessing the relationship between FALs and HRQoL, an HRQoL analysis cohort was comprised of those who completed the EQ-5D-5 L. Since HRQoL scores are continuous but bounded with an upper limit of 1, both ordinary least squares and tobit models were tested. All analyses were performed using STATA 17 (StataCorp LLC, College Station, TX, USA; www.stata.com).

## Results

### Study population

A total of 185 physicians and 1337 people with haemophilia were included in the CHESS data set, of whom 403 adult males with HA and no inhibitors receiving on-demand treatment were included in this study (Additional File Figure S1). The mean age was 37 years, 95% were White, and most PwHA were from Italy (29%), Spain (26%) and the UK (21%) (Table 1). The mean baseline FAL was 7.0 IU/dL (SD, 10.2). HA severity was fairly evenly distributed across the sample, with a slightly greater proportion of people with severe HA (Table 1). The mean ABR was 2.3 (SD, 2.64). A total of 167 PwHA completed the EQ-5D-5 L and were included in the HRQoL analysis cohort. The mean age of the participants included in the HRQoL cohort was 34 years, with a mean baseline FAL of 8.1 IU/dL (SD, 11.3) and mean ABR of 2.2 bleeds (SD, 2.4) (Table 1). Characteristics of the overall and HRQoL analysis cohorts were generally similar, though the HRQoL cohort was slightly younger.

**Table 1:** Baseline study population characteristics

	Overall (*N* = 403)	HRQoL analysis cohort (*N* = 167)^a^
Age		
Mean (SD) Median (range)	37.2 (16.0) 32 (18–87)	34.1 (14.7) 29 (18–6)
Race/ethnicity, *n* (%)		
White Other	383 (95) 20 (5)	161 (96) 6 (4)
Country, *n* (%)		
Italy France Germany Spain United Kingdom Romania	116 (29) 69 (17) 26 (7) 105 (26) 84 (21) 3 (< 1)	48 (29) 47 (28) 8 (5) 44 (26) 20 (12) 0 (0)
BMI, mean (SD)	24.5 (3.0)	24.6 (2.60)
Haemophilia severity, *n* (%)		
Mild (> 5–40%) Moderate (1–5%) Severe (< 1%)	121 (30) 131 (33) 151 (37)	53 (32) 53 (32) 61 (36)
Baseline FAL (% activity)		
Mean (SD) Median (range)	7.0 (10.2) 3.0 (0–40.0)	8.1 (11.3) 3 (0–40)
ABR		
Mean (SD) Median (range)	2.3 (2.6) 2.0 (0–15)	2.2 (2.4) 2 (0–14)
Chronic pain, *n* (%)		
None Mild Moderate Severe	158 (39) 150 (37) 79 (20) 16 (4)	65 (39) 68(41) 29 (17) 5 (3)
Problem joints, *n* (%)		
None 1 ≥2	282 (70) 81 (20) 40 (10)	117 (70) 33 (20) 17 (10)
Comorbidities, *n* (%)		
Anxiety Depression Type 2 diabetes mellitus Osteoarthritis Osteoporosis HIV Hepatitis B Hepatitis C	48 (12) 31 (8) 14 (4) 21 (5) 8 (2) 6 (2) 5 (1) 10 (3)	19 (11) 5 (3) 5 (3) 5 (3) 0 (0) 1 (< 1) 0 (0) 2 (1)
HRQoL, EQ-5D index score		
Mean (SD) Median (range)	–	0.78 (0.20) 0.77 (0.04–1.00)
HRQoL, EQ-5D VAS^a^		
Mean (SD) Median (range)	–	75.5 (16.8) 80.0 (25.0–100.0)

^a^EQ-5D index score was completed by 167 participants; EQ-5D VAS was completed by 165 participants

ABR, annual bleeding rate; BMI, body mass index; FAL, factor activity level; HIV, human immunodeficiency virus; HRQoL, health-related quality of life; SD, standard deviation; VAS, visual analog scale

### Regression analysis of FAL and ABR

**Fig. 1:**
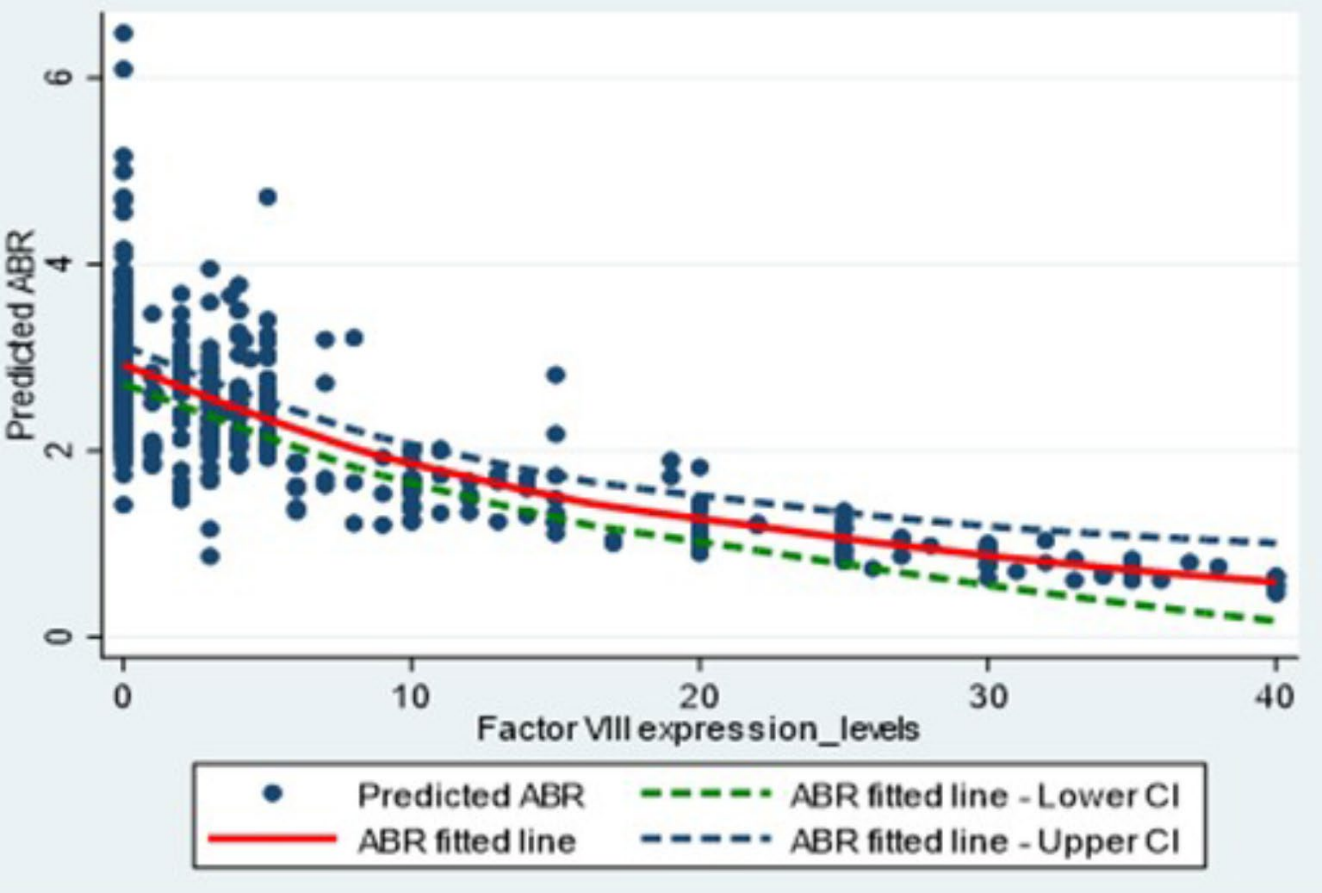
Predicted ABR from FALs (with 95% CIs). ABR, annual bleeding rate; CI, confidence interval; FAL, factor activity level

The negative binomial model (AIC, 1574.14) provided the best fit for modelling FAL with ABR. After adjusting for age, BMI, and presence of blood-borne diseases, which included HIV, HBV and HCV, the model showed that for every 1% increase in FAL, ABR decreased by 3.9% (0.09 events; *P* < 0.001; Additional File Table S1). The predicted number of bleeding events according to FAL showed a nonlinear relationship between FAL and ABR (Fig. 1). A relatively strong decrease in ABR was observed for FALs from 0 up to 10%, followed by a milder decrease for higher FALs. Table S2 in the supplement presents additional details on the distribution of FALs within the mild HA cohort and predicted ABR across differing FAL ranges (in 5% increments), decreasing by approximately 0.2 bleeds with each 5% increment in FAL.

### Regression analysis of FAL and HRQoL

**Fig. 2:**
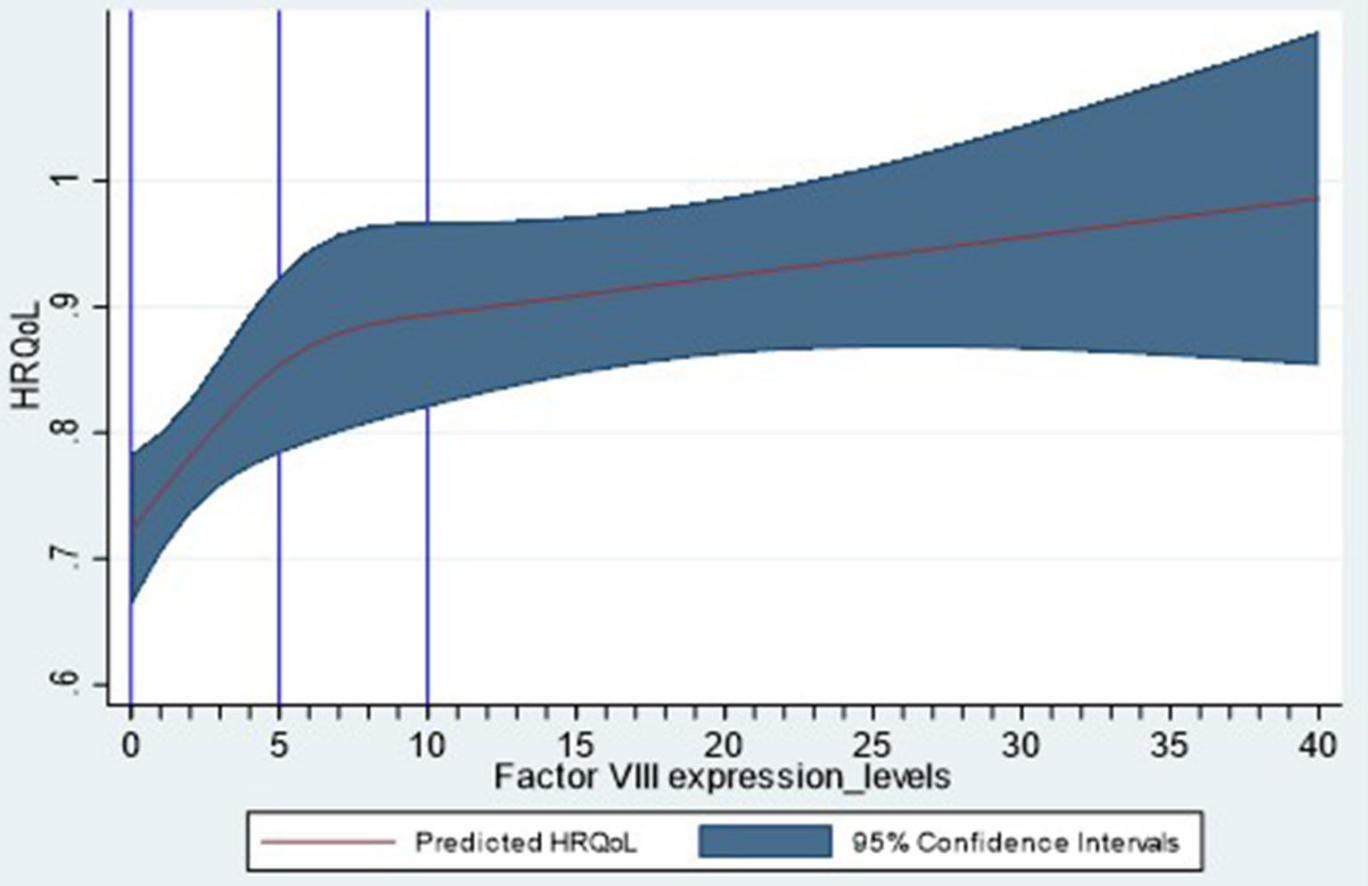
Predicted HRQoL from FALs (with 95% CIs). CI, confidence interval; FAL, factor activity level; HRQoL, health-related quality of life

The tobit model (AIC, 98.3) provided the best fit for modelling FAL and HRQoL. After adjusting for age, BMI, and presence of blood-borne viruses, the model showed that every 1% increase in FAL was associated with an increase of 0.0054 points in mean EQ-5D index score (*P* < 0.001; Additional File Table S3). A nonlinear relationship was observed between FAL and HRQoL, with a steep decrease in HRQoL score at FALs from 0 to 10% followed by a steadier decrease at higher FALs (Fig. 2).

## Discussion

This study found significant nonlinear relationships of FALs with ABR and HRQoL in a European cohort of adults with HA and no inhibitors. The negative binomial model suggested that an increase in average FAL of 1% was associated with a decrease in average ABR by 0.09. This would mean that, an average increase of 11.0% in FAL would be required to reduce ABR by 1. The tobit model suggested that average HRQoL (EQ-5D-5 L index score) would increase by 0.0054 points for every 1% increase in average FAL. Therefore, an increase in average FAL of 13% would be associated with a minimally important improvement in HRQoL scores of 0.07 points [22, 23].

These findings are consistent with those obtained from previous work among people with haemophilia B in the CHESS II and CHESS US studies [14]. In that work, when adjusting for age, BMI, and blood-borne viruses, a significant association between FALs and ABR was found, where a 1% increase in FAL was associated with an average 4.2% decrease in ABR (0.08 events) [14]. The analysis presented in this publication builds upon this existing work, and adds new information on a new cohort (PwHA). Understanding the relationship between FAL and ABR across levels of HA severity may further inform the interpretation of clinical trial findings. However, further research is needed to confirm the magnitude of ABR reductions associated with increases in FAL, as a range of results have been reported in the literature to date [21, 24]. Previous work explored the relationship between FAL and HRQoL among people with HB in the CHESS II and CHESS US studies, reporting a similar mean EQ-5D-5 L index score of 0.77 as observed in our HA cohort (mean 0.78 points) [15]. The model controlled for age, BMI and blood-borne viruses, and reported a 1% increase in FAL to be associated with an increase in mean EQ-5D-5 L index score of + 0.006 points (*P* = 0.003) [15]. Our study is consistent with prior work suggesting that ABR alone is not sufficient to inform clinical decisions in an evolving treatment landscape [25], emphasizing the need for more relevant measures and a multi-factor core outcome set [26].

Our findings should be considered in the context of certain strengths and limitations. These results should be generalizable to people with different levels of HA severity and different types of bleeds. However, the study cohort was recruited from European countries and our findings may not be as readily extended to those in other regions. A subset of the overall study population completed the EQ-5D-5 L questionnaire, which provided a smaller sample size for the FAL-HRQoL model. The HRQoL analysis population was generally similar to the overall study population, but further work to replicate this analysis using a larger data set would be beneficial. In addition, the relationship between HRQoL and FAL is non-linear meaning we could expect to see greater increases in HRQoL at very low FAL and smaller gains in HRQoL at higher FAL. It is important to consider the shape of the relationship between HRQoL and FALs as this can influence individual level results when compared to the average improvement in HRQoL due to an increase in FAL as reported in this study.

## Conclusions

To our knowledge, this study is the first to illustrate the relationship between FAL and ABR and HRQoL in PwHA. This study offers the first tangible estimates of how an increase in average FAL may be associated with lower ABR and better HRQoL, both of which are essential to shared patient-provider decisions, contextualizing emerging therapeutic options, and to health technology assessments. It is worthwhile to understand that increasing FALs not only benefits clinical but also humanistic outcomes.

## Electronic supplementary material

Below is the link to the electronic supplementary material.

Additional File 1: Detailed regression results tables and overview of participant attrition

## Data Availability

The data that support the findings of this study are available from HCD Economics but restrictions apply to the availability of these data, which were used under license for the current study, and so are not publicly available. Data are however available from the authors upon reasonable request and with permission of HCD Economics.

## Abbreviations

ABR: Annual bleeding rate
AIC: Akaike Information Criterion
BMI: Body mass index
CHESS: Cost of Haemophilia in Europe: a Socioeconomic Survey
FAL: Factor activity levels
FVIII: Factor VIII
HA: Haemophilia A
HBV: Hepatitis B virus
HCV: Hepatitis C virus
HIV: Human immunodeficiency virus
HRQoL: Health-related quality of life
IU: International Unit
PPIE: Patient Public Involvement Engagement
PwHA: People with haemophilia A
SD: Standard deviation
