# Supplementary material for: The association of factor VIII activity levels with bleeding and quality of life in haemophilia a: findings from the European CHESS II study
Source: Orphanet J Rare Dis. 2025 Jun 3;20:272. doi: 10.1186/s13023-025-03699-z (PMC12135601; doi:10.1186/s13023-025-03699-z)
Supplement: Supplementary file 1 — Additional File 1: Detailed regression results tables and overview of participant attrition [file 13023_2025_3699_MOESM1_ESM.docx]

SUPPLEMENT

**Table S1.** Negative binomial regression results for FAL and ABR (N=403)

|  | **Coefficients^a^** | **SE** | **Z** | **P>\|z\|** | **95% CI** |
| --- | --- | --- | --- | --- | --- |
| FAL | 0.961 | 0.005 | -7.01 | <0.001 | 0.950, 0.972 |
| Age | 0.999 | 0.003 | -0.29 | 0.774 | 0.993, 1.006 |
| BMI | 1.080 | 0.018 | 4.75 | <0.001 | 1.046, 1.115 |
| HIV | 0.940 | 0.422 | -0.14 | 0.891 | 0.390, 2.265 |
| HBV | 1.318 | 0.536 | 0.68 | 0.497 | 0.594, 2.923 |
| HCV | 0.893 | 0.314 | -0.32 | 0.747 | 0.448, 1.779 |
| Intercept | 0.436 | 0.182 | -1.99 | 0.046 | 0.193, 0.987 |
| alpha | 0.478 | 0.069 |  |  | 0.359, 0.635 |

ABR, annual bleeding rate; alpha: dispersion parameter; BMI, body mass index; CI, confidence interval; FAL, factor IX activity level; HBV, hepatitis B virus; HCV, hepatitis C virus; HIV, human immunodeficiency virus; SE, standard error.

^a^Incidence rate ratio

**Table S2.** Detailed FAL breakdown in mild HA cohort and respective predicted bleeding rates

|  | **Overall mild HA cohort^a^**  **(N=121)** |
| --- | --- |
| FAL 0-5%, N (%)  Predicted ABR, mean (SD) | 282 (70.0)  2.8 (0.7) |
| FAL >5-10%, n (%)  Predicted ABR, mean (SD) | 34 (28)  1.8 (0.5) |
| FAL 11-15%, n (%)  Predicted ABR, mean (SD) | 23 (19)  1.6 (0.4) |
| FAL 16-20%, n (%)  Predicted ABR, mean (SD) | 16 (13)  1.3 (0.3) |
| FAL 21-25%, n (%)  Predicted ABR, mean (SD) | 12 (10)  1.1 (0.2) |
| FAL 26-30%, n (%)  Predicted ABR, mean (SD) | 14 (12)  0.9 (0.1) |
| FAL 31-35%, n (%)  Predicted ABR, mean (SD) | 14 (12)  0.7 (0.1) |
| FAL 36-40%, n (%)  Predicted ABR, mean (SD) | 8 (7)  0.6 (0.1) |

^a^Reported proportions may not add to 100% due to rounding

**Table S3.** Tobit model regression results for FAL and HRQoL (N=167)

|  | **Coefficients^a^** | **SE (delta)** | **Z** | **P>\|z\|** | **95% CI** |
| --- | --- | --- | --- | --- | --- |
| FAL | 0.0054 | 0.0013 | 4.08 | <0.001 | 0.0028, 0.0080 |
| Age | -0.0014 | 0.0010 | -1.36 | 0.172 | -0.0035, 0.0006 |
| BMI | -0.0098 | 0.0060 | -1.64 | 0.010 | -0.0215, 0.0019 |
| HIV | 0.2005 | 0.0411 | 4.87 | <0.001 | 0.1198, 0.2811 |
| HCV | -0.3467 | 0.2283 | -1.52 | 0.129 | -0.7942, 0.1010 |

BMI, body mass index; CI, confidence interval; FAL, factor IX activity level; HCV, hepatitis C virus; HIV, human immunodeficiency virus; SE, standard error.

^a^Average marginal effects at HIV, HCV=0 holding other variables constant.

**Figure S1.** Participant attrition


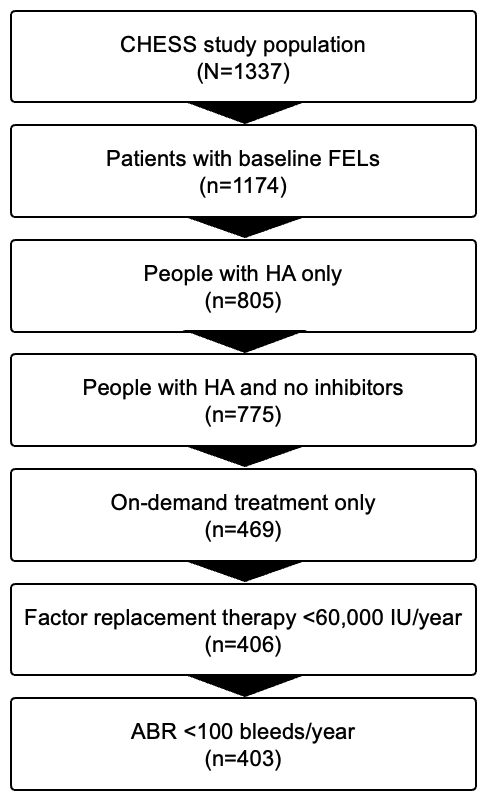


ABR, annual bleeding rate; FAL, factor activity level; HA, haemophilia A.
